# Does mid-luteal progesterone predict pregnancy in intrauterine insemination cycles following sequential clomiphene citrate and gonadotropin treatment?

**DOI:** 10.1097/MD.0000000000034754

**Published:** 2023-09-01

**Authors:** Mehmet Çinar, Aytekin Tokmak, Meryem Kuru Pekcan, Mustafa Sarsu, Yaprak Üstün, Gülnur Özakşit

**Affiliations:** a Zekai Tahir Burak Women’s Health Research and Education Hospital, Obstetrics and Gynecology, Ankara, Turkey.

**Keywords:** clomiphene, gonadotropin, intrauterin insemination, luteal phase, progesterone

## Abstract

This study aimed to determine whether serum mid-luteal progesterone (MLP) levels measured in the current treatment cycles of infertile women undergoing controlled ovarian hyperstimulation and intrauterine insemination following the sequential use of clomiphene citrate and gonadotropin may predict pregnancy. A total of 107 consecutive anovulatory women were included in this prospective cohort study. Patients with other causes of infertility were also excluded from the study. None of the patients received progesterone treatment for luteal phase support. The data recorded for each woman included age, body mass index, infertility type and duration, basal hormone levels, and previous and current cycle characteristics with MLP levels. Ovulation was confirmed using MLP and sonographic evaluation in all patients. An MLP level of > 3 ng/mL was regarded as a sign of ovulation. After treatment, the patients were divided into 2 groups according to the presence or absence of pregnancy, and the obtained data were compared between the groups. There were no significant differences in age, body mass index, or basal hormone levels between the 2 groups (all *P* > .05). However, the duration of infertility was significantly shorter in the pregnancy group (*P* = .003). The anovulation rate in this cohort was 18.7% (n = 20). A total of 15 (14%) were examined. MLP levels were 25.1 ± 13.8 ng/mL and 18.3 ± 14.5 ng/mL in the pregnant and nonpregnant groups, respectively (*P*:.089). Based on the receiver operating characteristic curve analysis, it was determined that there was no predictive value of the mid-luteal phase progesterone level for pregnancy in patients in whom ovulation was detected. Mid-luteal serum progesterone levels did not predict pregnancy in infertile women who underwent controlled ovarian hyperstimulation with sequential clomiphene citrate plus gonadotropin treatment and intrauterine insemination.

## 1. Introduction

Controlled ovarian hyperstimulation (COH) and intrauterine insemination (IUI) are generally used as the first step of treatment in couples with infertility due to ease of procedure and cheaper costs.^[1]^ IUI is one of the oldest assisted reproductive technologies, but it is still widely used in COH cycles.^[2]^ Although the goal is to develop a maximum of 1 or 2 follicles during COH, sometimes supraphysiological hormone levels can impair implantation and reduce treatment success.

Progesterone (P4) is a steroidal hormone that plays prominent roles in ovulation, menstruation, fertilization, implantation, and embryogenesis. Although the main source of P4 secretion is the corpus luteum (CL), P4 is also synthesized in the ovarian follicles, adrenal cortex, brain, and placenta.^[3,4]^ CL incurs luteolysis and loses the ability to produce P4 by the end of the menstrual cycle; therefore, serum P4 concentration reaches its lowest levels in the early follicular phase if it is not supported by the production of human chorionic gonadotropin (hCG) secreted from placental tissues.^[5,6]^

P4 regulates the implantation process, immune responses during pregnancy by releasing certain anti-abortive cytokines and inhibits myometrium contractions till the end of pregnancy.^[7]^ With the implantation formation, hCG starts to be secreted by cyto/syncytiotrophoblast and interacts with the luteinizing hormone (LH)/hCG receptor of the ovary and promotes the maintenance of the corpus luteal P4 production. Thereafter, the placenta takes over P4 production from the CL when sufficient syncytiotrophoblast cells are present in the uterus. P4 deficiency or usage of progesterone receptor antagonist before the first 7 weeks of pregnancy causes miscarriages.^[8]^ There is a general perception that higher levels of mid-luteal progesterone (MLP) may be associated with higher pregnancy rates. This perception is based on the idea that high progesterone levels may be associated with the quality and quantity of follicles and oocytes in COH-IUI cycles.^[9,10]^

Many studies have evaluated predictive factors for pregnancy after COH-IUI cycles were conducted previously. Both dysfunctional and short luteal phase lengths have been reported following COH with gonadotropins (GTs) or clomiphene citrate (CC).^[11,12]^ However, the link between hormonal status during the luteal phase and pregnancy outcomes in COH-IUI cycles remains unclear. This might be due to the lack of a universal definition of luteal phase insufficiency and knowledge about its prevalence and clinical significance.

This study was designed to determine whether serum MLP levels measured in the present COH–IUI cycle with sequential use of CC and GTs might predict pregnancy.

## 2. Material and methods

The study was conducted at the infertility outpatient clinics of Dr. Zekai Tahir Burak Women Health Research Hospital between January 2017 and July 2017. The study protocol was approved by the Ethical Committee of the hospital, and written informed consent was obtained from each woman.

In total, 107 consecutive women with anovulatory infertility were recruited for this prospective cohort study. Diagnostic evaluation of infertility was made after the following investigations: (i) menstrual cycles longer than 35 days and normal or higher basal antral follicle count and ovarian volume on transvaginal sonogram equipped with a 5 MHz transvaginal probe (Logiq 200 Pro, General Electric, Milwaukee, WI); (ii) normal endometrial cavity and tubal patency on hysterosalpingography; (iii) no adnexal or uterine pathology on transvaginal sonogram; (iv) normal hormone parameters on cycle day 3, including serum estradiol (E2), follicle-stimulating hormone (FSH), LH, thyroid-stimulating hormone, prolactin, and 17 hydroxy progesterone measured by ELISA; and (v) normal spermiogram parameters based on World Health Organization criteria.^[13]^ World Health Organization class II anovulation patients, the majority of which were formed in PCOS cases, were included in the study. PCOS was diagnosed using the Rotterdam criteria of ESHRE-ASRM.^[14]^ Chronic anovulation was confirmed by displaying a serum level of progesterone <3 ng/mL during the mid-luteal phase. Women with endocrine diseases such as thyroidopathies, diabetes mellitus, Cushing syndrome, or other chronic systemic diseases, and those with a history of adnexal or uterine surgery were excluded. Patients who had pathological findings in hysterosalpingography, whose partners had insufficient sperm parameters for IUI, and women older than 40 years or younger than 20 years of age were also excluded from the study.

The data recorded for each woman included age, body mass index (BMI), infertility type, etiology and duration, basal hormone levels, and previous and current cycle characteristics with MLP levels. After treatment, the patients were divided into 2 groups according to the presence or absence of pregnancy, and the obtained data were compared between the groups. An MLP level of >3 ng/mL was regarded as a sign of ovulation. The clinical characteristics and treatment success rates of the patients were compared.

### 2.1. COH and IUI protocols

The treatment protocols for each patient were individualized according to age, BMI, ovarian appearance, serum FSH and E2 values, history, and response to previous treatments. All patients underwent IUI after CC and GT for COH. CC (50–150 mg/d, per oral) was administered for 5 consecutive days between days 2 and 6 of the cycle, followed by the administration of recombinant-FSH (37.5–75 IU) on the last day of CC treatment. Ovarian follicular responses were monitored using serum E_2_ levels and ultrasonography after day 6 of stimulation and every 2–3 days thereafter. Preovulatory follicle development was monitored until at least 1 follicle was 17–22 mm in diameter. Recombinant hCG (HCG 250 µg) was administered subcutaneously. A single IUI under ultrasound guidance was performed in all patients 34–36 hours after hCG administration. The sperm samples were processed using the swim-up method in an andrology laboratory. Seven days after the IUI procedure, blood samples were collected for serum P4 measurements, and serum hCG levels were measured on day 14 of IUI. Clinical pregnancy was defined as the presence of an intrauterine gestational sac and/or fetal cardiac activity. None of the patients had received progesterone treatment for luteal phase support until the assay was performed.

### 2.2. Statistical analysis

Statistical analyses were performed using SPSS version 22 (Statistical Package for the Social Sciences, Chicago, IL). Student *t*-test was performed for continuous variables, which were normally distributed and the distributions have been tested using the Shapiro–Wilk test. Mann–Whitney has been performed for continuous variables that were not normally distributed and the chi-square test has been done for nonparametric variables between the groups. Receiver operating characteristic curve analysis was used to evaluate the performance of early follicular phase serum P4 concentration in predicting clinical pregnancy. *P* was set at *P* < .05.

## 3. Results

This study included 107 initial COS + IUI cycles for patients treated with sequential CC and GTs. During the study period, 1 patient experienced a biochemical pregnancy, whereas 15 pregnancies continued as a clinical pregnancy. The clinical pregnancy rate in this cohort was 14%. The groups were similar in terms of age, BMI, primary infertility rate, and infertility etiology. The infertility time was significantly shorter in the pregnant group than in the nonpregnant group (*P* < .05) (Table 1). Similarly, no significant differences were observed between the groups in baseline hormone levels (*P* > .05).

**Table 1:** Comparison of demographics, laboratory parameters, and cycle characteristics between the groups.

Demographic features	Pregnant (n:15)	Nonpregnant (n:92)	*P*
Age (years)	27.2 ± 2.5	29.1 ± 4.8	.140
BMI (kg/m^2^)	24.0 ± 2.5	24.6 ± 2.8	.416
Primary infertility n (%)	13 (86.7)	76 (82.6)	.697
Infertility time (years)	3.1 ± 1.8	5.1 ± 3.0	**.003**
Infertility etiology			.647
Anovulatory n (%)	4 (26.7)	30 (32.6)	
PCOS n (%)	11 (73.3)	62 (67.4)	
*Baseline hormone levels*			
Estradiol (pg/mL)	32.5 ± 18.0	40.9 ± 16.2	.069
FSH (U/L)	8.0 ± 2.1	7.0 ± 1.8	.054
LH (U/L)	9.1 ± 4.1	8.5 ± 3.3	.570
Prolactin (ng/dL)	9.8 ± 4.2	10.2 ± 6.0	.766
TSH (U/L)	2.5 ± 0.9	2.2 ± 1.2	.349
*Cycle characteristics*			
Midluteal P (ng/mL)	25.1 ± 13.8	18.3 ± 14.5	.089
Ovulation (*P* > 3 ng/mL) n (%)	15 (100%)	72 (78.3%)	**.045**
HCG trigger day	13.2 ± 4.5	13.5 ± 3.5	.389
HCG day P (ng/mL)	0.7 ± 1.1	0.5 ± 0.3	.899
HCG day E2 (pg/mL)	553.6 ± 383.6	470.0 ± 289.5	.533
Endometrial thickness (mm)	9.0 ± 1.8	8.4 ± 2.2	.340
Cc dose (mg)	100 (50–150)	50 (50–150)	.687
Initial GT dose (IU)	37.5 (37.5–75)	37.5 (37.5–75)	.809
Total GT dose (IU)	478.3 ± 578.9	431.1 ± 446.8	.494
Prior CC cycle	3 (1–6)	3 (1–7)	.480
Previous cycle cancellation	0 (0–1)	0 (0–2)	.386
Prior IUI application	3 (1–5)	3 (1–8)	.340
RO follicle number ≥ 14 mm			.663
0	3 (20)	24 (26.1)	
1	11 (73.3)	56 (60.9)	
2	1 (6.7)	11 (12)	
LO follicle number ≥ 14 mm			.592
0	8 (53.3)	40 (43.5)	
1	6 (40)	48 (52.2)	
2	1 (6.7)	3 (3.3)	
LO follicle number ≥ 18 mm			.136
0	9 (60)	60 (65.2)	
1	5 (33.3)	31 (34.8)	
2	1 (6.7)	0	
RO follicle number ≥ 18 mm			.792
0	8 (53.3)	43 (47.8)	
1	7 (46.7)	46 (50)	
2	0	2 (2.2)	

Data were expressed as mean ± standard deviation, median (minimum–maximum), and number (percentage) where applicable. A *P* value > .05 was considered as statistically significant.

BMI = body mass index, CC = clomiphene citrate, FSH = follicle stimulating hormone, GT = gonadotropin, HCG = human chorionic gonadotropin, IUI = intrauterine insemination, LH = luteinizing hormone, LO = left ovary, P = progesterone, PCOS = polycystic ovary syndrome, RO = right ovary, TSH = thyroid-stimulating hormone.

**Figure 1.**
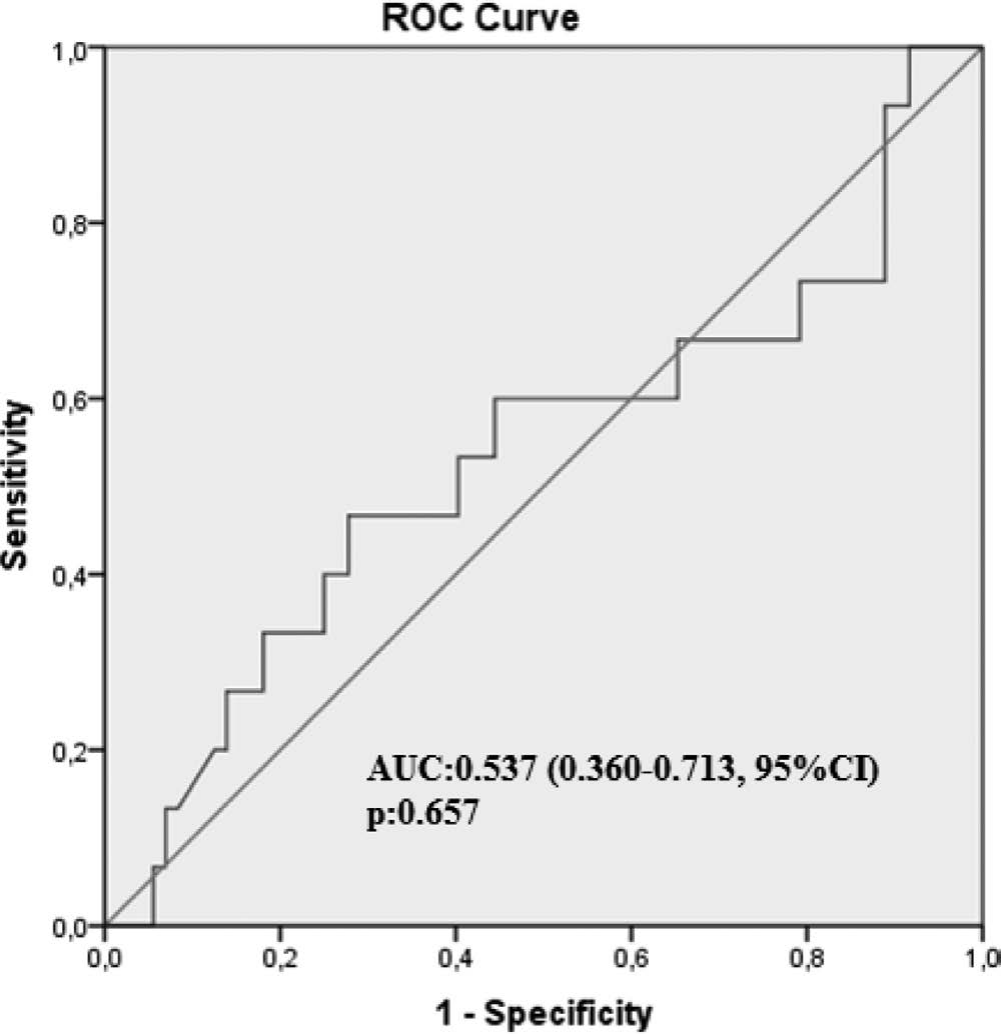
ROC curve analysis of MLP level in predicting pregnancy in the patients whom ovulation was detected. MLP = mid-luteal progesterone, ROC = receiver operating characteristic.

The anovulation rate, as determined using the MLP value, was 18.7% (20 patients). MLP levels were 25.1 ± 13.8 and 18.3 ± 14.5 ng/mL in the pregnant and nonpregnant groups, respectively (*P*: .089). In further analysis, mean MLP values of ovulated women was calculated as 23.2 ± 12.7 ng/mL in the nonpregnant group. Receiver operating characteristic curve analysis also showed that there was no predictive value for pregnancy in patients in whom ovulation was detected (Fig. 1).

The patients’ previous and current cycle characteristics are listed in Table 1. No cycle cancelation occurred in any patient during COH treatment. There were no statistically significant differences in the clinical, ultrasonographic, and hormonal parameters between the groups, although some good prognostic factors, such as endometrial thickness and E2 levels on HCG day, were slightly higher in the pregnant group. The number of leading follicles was also similar between the 2 groups (*P* > .05).

## 4. Discussion

First-line therapy in anovulatory couples usually consists of ovulation induction with CC followed by sexual intercourse or IUI. Next, GT was used in conjunction with IUI treatment. The sequential use of CC and GT with IUI is rarely performed, although it has advantages such as reduced cost, greater tolerability than GT treatment alone, and satisfactory results, particularly in CC-resistant infertile women.^[15]^ In this study, we sought to determine whether serum MLP level, which has never been studied in women undergoing COH + IUI with the use of CC plus GT treatment, predicts clinical pregnancy. According to our results, MLP did not predict pregnancy in infertile anovulatory women.

Although it is well known that COH interacts in many ways with the luteal phase, the luteal phase endocrine profile in COH cycles has rarely been monitored, particularly in IUI cycles. After COH with GTs, supraphysiological luteal phase progesterone and estradiol concentrations inhibit pituitary LH secretion, which causes demise of the CL, withdrawal of bleeding, and a low chance of pregnancy. These effects are rarely observed in the COH + IUI cycles, where minimal stimulation is performed. However, it does not change the fact that P4 is an important hormone for the continuation of pregnancy.

Historically, there is a general perception that higher MLP concentrations are associated with better treatment outcomes in GT- or CC-induced cycles.^[16–19]^ Conversely, Costello et al^[9]^could not identify a relationship between MLP concentration and clinical pregnancy rate in COH-IUI treatments with GTs, but they suggested that a low level may predict treatment failure. Similarly, Hansen et al^[19]^ found that a low MLP concentration is associated with a low probability of live births during COH-IUI treatment, particularly in women treated with GTs. A recent meta-analysis showed that progesterone support did not benefit patients undergoing COH with CC or CC plus GTs, suggesting CC provides a better luteal phase function.^[20]^ However, luteal phase support with progesterone was found to be beneficial to patients undergoing ovulation induction with GTs in IUI cycles.

The number of mature follicles increases the likelihood of a pregnancy. It has been suggested that higher concentrations of MLP are associated with the formation of more than 1 CL, as a consequence of an increased number of mature follicles. Currently, owing to the increased risk of ovarian hyperstimulation and multiple pregnancies, 1 or 2 follicle developments are targeted using mild stimulation protocols. It has been suggested that the cycle should be canceled or converted to IVF in the presence of excessive preovulatory follicles. MLP concentrations were also found to be inversely associated with BMI in a previous study.^[11]^ The reduced levels of MLP in obese women were most likely attributed to predominant monofollicular development rather than CL deficiency. In our study, there were no significant differences between the groups with regard to the mean BMIs and number of mature follicles.

The predictive ability of mid-luteal estradiol and progesterone levels for ongoing pregnancy has been found to be meaningful in IVF–ICSI cycles.^[21]^ However, hormonal alterations during the luteal phase have not been investigated in IUI cycles in detail. A recent study performed on unexplained infertile women undergoing GT and IUI treatment showed that the addition of the % change of progesterone between days 6 and 10 following the hCG trigger did not improve the predictive ability provided by the change in estradiol.^[10]^

The different results reported in the literature are due to the fact that the patient groups are not homogeneous, hormonal evaluations are performed on different days after the HCG trigger, and methods and evaluation of hormone assays are not standard. Although our study has some limitations, such as the small sample size, this is the first study to evaluate the role of MLP levels in predicting pregnancy in patients with infertility who underwent sequential treatment.

In conclusion, MLP serum P4 level could not predict clinical pregnancy in IUI cycles following the sequential use of CC plus GT. It also seems that the sequential use of CC and GTs ensures a sufficient luteal phase in women with anovulatory infertility. Therefore, we conclude that routine screening for MLP is not effective for early prediction of treatment outcomes in women who have undergone IUI cycles following the sequential use of CC plus GT. Our findings are compatible with a recent meta-analysis that showed that progesterone support did not benefit patients undergoing IUI and COH with CC plus GTs. However, this finding requires prospective validation prior to clinical implementation.

## Author contributions

**Conceptualization:** Mehmet Çinar, Aytekin Tokmak, Yaprak Üstün, Gülnur Özakşit.

**Data curation:** Mehmet Çinar, Aytekin Tokmak, Meryem Kuru Pekcan, Mustafa Sarsu.

**Formal analysis:** Mehmet Çinar, Aytekin Tokmak, Gülnur Özakşit.

**Funding acquisition:** Yaprak Üstün.

I**nvestigation:** Mehmet Çinar, Aytekin Tokmak, Meryem Kuru Pekcan, Mustafa Sarsu, Gülnur Özakşit.

**Methodology:** Aytekin Tokmak, Meryem Kuru Pekcan, Mustafa Sarsu.

**Project administration:** Aytekin Tokmak, Meryem Kuru Pekcan, Yaprak Üstün, Gülnur Özakşit.

**Resources:** Mehmet Çinar, Mustafa Sarsu, Yaprak Üstün, Gülnur Özakşit.

**Software:** Mehmet Çinar, Aytekin Tokmak, Meryem Kuru Pekcan, Mustafa Sarsu.

**Supervision:** Aytekin Tokmak, Yaprak Üstün, Gülnur Özakşit.

**Visualization:** Mehmet Çinar, Aytekin Tokmak, Meryem Kuru Pekcan, Yaprak Üstün.

**Writing – original draft:** Mehmet Çinar.

**Writing – review & editing:** Aytekin Tokmak.
